# RNA binding protein YWHAZ mediates specific mRNA translation and regulates cell proliferation and apoptosis in diabetic foot ulcer

**DOI:** 10.3389/fmed.2026.1751279

**Published:** 2026-05-07

**Authors:** Tianjian Zha, Junjie Yao, Hao Wang, Qiang Cao, Jian Zhang, Zhao Chen, Jie Wang

**Affiliations:** Department of Burns, Wound Repair Surgery, People’s Hospital of Xinjiang Uygur Autonomous Region, Urumqi, Xinjiang, China

**Keywords:** diabetic foot ulcer, iRIP-seq, proliferation, SREBF1, YWHAZ

## Abstract

**Background:**

Diabetic foot ulcer (DFU), a condition marked by high rates of recurrence, amputation, and mortality, represents one of the major challenges in diabetes management. RNA-binding proteins (RBPs) are pivotal for post-transcriptional regulation in diabetic complications. However, the aberrantly expressed RBP genes and their regulatory mechanisms in DFU remain unclear. This study aimed to investigate the potential functions and molecular interactions of YWHAZ, a dysregulated RBP identified in DFU tissues.

**Methods:**

YWHAZ was selected for further investigation based on its dysregulation in DFU tissues as identified through an independent public RNA-seq dataset. Reverse transcription quantitative polymerase chain reaction (RT-qPCR) and immunohistochemistry were used to validate YWHAZ expression. The biological behavior of HaCaT cells with YWHAZ knockdown (Si_YWHAZ) was compared with that of control cells. Differentially expressed genes (DEGs) were identified by RNA-seq. Additionally, improved RNA immunoprecipitation (iRIP)-seq was employed to investigate potential binding interactions of YWHAZ in DFU tissues.

**Results:**

YWHAZ was significantly upregulated and validated as an RBP in DFU by RT-qPCR and immunohistochemistry. Cellular experiments revealed that Si_YWHAZ facilitated proliferation and migration while inhibiting apoptosis, consistent with its upregulation in DFU. RNA-seq identified 1,072 DEGs in Si_YWHAZ cells. Upregulated genes were significantly enriched for cell proliferation-related processes and included *AREG*, *FOSL1*, *HAS2,* and *IL7R*, whereas downregulated genes were associated with cell adhesion, including *LAMB3*, *SLAMF7*, *COL12A1,* and *ITGA5*. iRIP-seq results demonstrated that YWHAZ interacts with a large number of mRNAs and is located in the CD and intron region of the genome. Furthermore, the ABLIFE algorithm indicated that YWHAZ binds to GC-rich motifs. Integrated iRIP-seq and RNA-seq analyses identified 57 DEGs that were selectively bound by YWHAZ, most of which were downregulated. Notably, *SREBF1*, which is positively associated with type 2 diabetes risk. KEGG pathway analysis revealed that *SREBF1* is enriched in both the insulin resistance and insulin signaling pathways.

**Conclusion:**

The study results suggest the cellular functions and molecular targets of YWHAZ, indicating its potential regulatory role in DFU development. This study provides valuable insights into the regulatory mechanisms of YWHAZ in DFU for future investigations.

## 1 Introduction

Diabetes is becoming a rapidly escalating health crisis worldwide in the 21st century. According to the International Diabetes Federation in 2021, approximately 536.6 million adults were affected by the condition. Globally, approximately 18.6 million people with diabetes develop a foot ulcer every year (1). Diabetic foot ulcer (DFU), a frequent and serious complication, poses substantial challenges—not only to patients, in the form of hospitalization, amputation, and disability, but also for society through increased healthcare resource demands and economic costs (2). The mammalian body has evolved a carefully orchestrated series of defenses against skin wounding, which include inflammation, cell proliferation, differentiation, and migration (3). The epidermis plays a vital role in the healing of skin wounds, with the proliferative phase being characterized by epidermal cell proliferation and migration into the wound bed, thereby facilitating wound closure (4). Keratinocytes, as the main cellular component of the epidermis, exhibit reduced proliferation and impaired migration in diabetic wounds (5). Previous studies have demonstrated that hyperglycemia is a driving factor in the pathology of impaired wound healing in diabetes. Even short-term exposure (24 or 72 h) to high glucose levels (25 mM), representing an acute effect of hyperglycemia, significantly compromises keratinocyte migration (6). Consequently, investigating the mechanisms that promote DFU healing is essential for the development of novel therapeutic strategies.

RNA-binding proteins (RBPs) are central mediators of post-transcriptional gene regulation, governing nearly all aspects of RNA metabolism and function (7). In recent years, numerous RBPs have been shown to play significant roles in a wide range of human diseases, including cardiovascular disorders, endocrine dysfunction, cancer, and neurodegenerative conditions (8–10). As research into the pathogenesis of DFU deepens, attention has increasingly turned to the potential involvement of RBPs in this condition (11). YWHAZ (also known as 14–3-3ζ) functions as a central hub protein in numerous signal transduction pathways and plays a critical role in disease progression. For instance, modulating intestinal YWHAZ expression has been proposed as a therapeutic strategy to enhance circulating GLP-1 levels and improve glucose homeostasis (12). In diabetic nephropathy, microRNA-451 negatively regulates the expression of YWHAZ to inhibit the progression of diabetic nephropathy (13). Despite these insights, the specific role and post-transcriptional regulatory mechanisms of YWHAZ in the impaired healing process of DFU remain largely unexplored. Our research aims to address this gap.

RNA-sequencing (RNA-seq) has emerged as a widely adopted tool for delineating aberrant transcriptome profiles in diabetes research. For instance, previous studies using RNA sequencing have revealed that genes *BCL2* and *FOXP2*, which are related to oxidative stress, can serve as diagnostic markers for DFU (14). Hu et al. (15) revealed that all DFU tissues exhibited widespread upregulation of immune activation genes by RNA-seq. In addition, RNA-seq revealed many activated and inhibited transcription factors and microRNAs associated with non-healing chronic DFUs in humans (16).

In our study, we hypothesized that YWHAZ, as a critical RBP, is dysregulated in non-healing DFU and contributes to impaired keratinocyte function under high-glucose conditions through specific post-transcriptional regulatory networks. To verify this, we performed RNA-seq analysis and found that YWHAZ was highly expressed in ulcerated skin tissues from patients with non-healing DFU. RT-qPCR and immunohistochemistry results showed that YWHAZ was upregulated in non-healing DFU tissues. Subsequently, we adopted a high-glucose model combined with RNA-seq and improved RNA immunoprecipitation and sequencing (iRIP-seq) to explore the binding mode of YWHAZ in DFU. These findings provide valuable insights into DFU pathogenesis and lay the foundation for identifying novel diagnostic markers and therapeutic targets.

## 2 Materials and methods

### 2.1 Patients and sample collection

Patient ulcerated skin tissues (*n* = 7) and control skin tissues from healthy individuals (*n* = 7) were procured for this study. The inclusion of human tissue samples enhances the clinical relevance of the study, although the sample size is limited. Each sample was collected and preserved in RNA preservation solution at −80 °C. All participating patients provided written informed consent. All experiments were performed in accordance with the relevant institutional guidelines and regulations.

### 2.2 Cell culture and transfection

HaCaT cells were obtained from Wuhan Pricella Biotechnology Co., Ltd. The identity of the HaCaT cell line was authenticated using short tandem repeat (STR) analysis. The cell line was cultured at 37 °C with 5% CO_2_ in basal medium supplemented with 10% fetal bovine serum (FBS) (10091148, Gibco, USA), 100 μg/mL of streptomycin, and 100 U/mL of penicillin (SV30010, HyClone, USA). The YWHAZ siRNAs and negative control (NC) sequences were transfected into the indicated cells using Lipofectamine™ RNAiMAX Transfection Reagent (13778030, Invitrogen, USA), following the manufacturer’s instructions.

YWHAZ siRNAs-forward: (5′-GCACGCUAAUAAUGCAAU UTT-3′).

YWHAZ siRNAs-reverse: (5′-AAUUGCAUUAUUAGCGUG CTT-3′).

SiRNA NC-forward: (5′-UUCUCCGAACGUGUCACGUTT-3′).

siRNA NC-reverse: (5′-ACGUGACACGUUCGGAGAATT-3′).

### 2.3 Western blot

The HaCaT cells were lysed in ice-cold radio-immunoprecipitation assay (RIPA) buffer (PR20001, Proteintech, China) supplemented with a protease inhibitor cocktail (4693116001, Sigma, USA) and incubated on ice for 30 min. Samples were boiled for 10 min in protein loading buffer (P1040, Solarbio, China) in a boiling water bath, then loaded onto a 10% sodium dodecyl sulfate-polyacrylamide gel electrophoresis (SDS-PAGE) gel and transferred to 0.45 mm polyvinylidene difluoride (PVDF) membranes (ISEQ00010, Millipore, USA). The PVDF membranes were incubated for 1 h at room temperature and incubated overnight at 4 °C with primary antibodies: YWHAZ (1:1,000, PA5-27317, THERMO, USA) and *β*-tubulin (1:1,000, 10094-1-AP, Proteintech, China). This was followed by an incubation with horseradish peroxidase-conjugated secondary antibodies (anti-rabbit, 1:10,000, SA00001-2, Proteintech, China; or anti-mouse, 1:10,000, AS003, ABclonal, China) for 45 min at room temperature. Then, the membranes were visualized using the enhanced ECL reagent (P0018FM, Beyotime, China) through chemiluminescence.

### 2.4 Real-time qPCR validation

cDNA synthesis was performed using a reverse transcription kit (R323-01, Vazyme, China) at 42 °C for 5 min, 37 °C for 15 min, and 85 °C for 5 s on the MyCycler (T100, Bio-Rad, USA). Furthermore, qPCR was performed on the ABI QuantStudio 5, with denaturation at 95 °C for 10 min, followed by 40 cycles of denaturation at 95 °C for 15 s and annealing and extension at 60 °C for 1 min. Each sample was run in replicates. The concentration of each transcript was then normalized to glyceraldehyde-3-phosphate dehydrogenase (GAPDH), and mRNA levels were calculated using the 2^−ΔΔCT^ method (Livak and Schmittgen, 2001). Statistical comparisons were performed using two-way ANOVA with GraphPad Prism software (Version 8.0, San Diego, CA, USA).

YWHAZ-forward: (5′-TTGCCGCTGGTGATGACA-3′).

YWHAZ-reverse: (5′-CAGTCTGATAGGATGTGTTGGT-3′).

Hum-GAPDH-forward: (5′-TGGTAGAAGGTAGTGGGTA GAA-3′).

Hum-GAPDH-reverse: (5′-GAGGTAGAGTTGGAAAGGG AAG-3′).

### 2.5 Immunohistochemistry

Ulcerated skin tissues from DFU patients and normal control tissues were fixed in 4% paraformaldehyde for 24 h, followed by routine dehydration and paraffin embedding. Tissues were sectioned at 5 μm using a microtome and mounted on poly-L-lysine-coated slides. Subsequently, antigen retrieval was performed by heating the slides in citrate buffer (pH 6.0) (10007118, Sinopharm, China) at 100 °C for 5 min using an autoclave. After cooling to room temperature, the sections were blocked with 10% normal serum to reduce non-specific binding. Slides were then incubated overnight at 4 °C with the YWHAZ antibody at a dilution of 1:200. The following day, the slides were washed and incubated with secondary antibodies for 30 min. Freshly prepared 3,3′-diaminobenzidine (DAB) (100 μL; DAB4033, Maxim, China) was applied to each slice and observed under a microscope. The slides were counterstained with hematoxylin (BA4041, BaSO, China), dehydrated, cleared, and mounted for microscopic examination. The number of clinical samples used for immunohistochemistry and the relevant ulcer characteristics are shown in Table 1.

**Table 1:** The number of clinical samples used for immunohistochemistry and the relevant ulcer characteristics.

Number	Duration of diabetes (years)	Age	Gender	Location of the ulcer	Ulcer characteristics
BB1	—	67	Male	The toe of the right foot	Full-thickness skin
BB2	—	21	Male	Left leg	Full-thickness skin
BB3	—	57	Female	Dorsum of left foot	Partial-thickness skin (not extending into the dermis)
BB4	—	67	Female	Plantar surface of the left foot	Full-thickness skin
BB5	—	61	Female	Chest	Full-thickness skin
BB6	—	48	Male	Neck	Partial-thickness skin (not extending into the dermis)
BB7	—	75	Female	The toe of the left foot	Full-thickness skin
BB8	—	57	Male	Heel of the left foot	Full-thickness skin
BB9	—	69	Female	Chest	Full-thickness skin
BB10	—	35	Male	Dorsum of the left foot	Full-thickness skin
BB11	—	20	Male	Dorsum of the right foot	Partial-thickness skin (not extending into the dermis)
BB12	—	55	Female	Sole of the right foot	Full-thickness skin
BB13	—	62	Male	Dorsum of the right foot	Full-thickness skin
BB14	—	54	Female	Abdomen	Partial-thickness skin (not extending into the dermis)
BB15	—	18	Male	Chest	Partial-thickness skin (not extending into the dermis)
BB16	—	62	Male	Fingers of the right hand	Full-thickness skin
AA1	22	78	Male	Medial malleolus of the left foot	Wagner Grade IV ulcer with full-thickness skin involvement but without bone exposure
AA2	13	53	Male	Toes of the right foot	Wagner Grade IV ulcer with full-thickness skin involvement but without bone exposure
AA3	21	69	Male	Toes of the left foot	Wagner Grade IV ulcer with full-thickness skin involvement but without bone exposure
AA4	18	66	Male	Dorsum of the left foot	Wagner Grade IV ulcer with full-thickness skin involvement but without bone exposure
AA5	7	61	Male	Toes of the right foot	Wagner Grade IV ulcer with full-thickness skin loss and bone exposure
AA6	12	55	Male	Toes of the right foot	Wagner Grade IV ulcer with full-thickness skin involvement but without bone exposure
AA7	17	56	Female	Toes of the left foot	Wagner Grade IV ulcer with full-thickness skin involvement but without bone exposure
AA8	30	47	Male	Sole of the left foot	Wagner Grade IV ulcer with full-thickness skin loss and bone exposure
AA9	1	74	Female	Toes of the left foot	Wagner Grade IV ulcer with full-thickness skin involvement but without bone exposure
AA10	12	56	Male	Lateral malleolus of the left foot	Wagner Grade IV ulcer with full-thickness skin loss and bone exposure
AA20	14	73	Male	Toes of the right foot	Wagner Grade IV ulcer with full-thickness skin involvement but without bone exposure
AA12	17	55	Male	Toes of the left foot	Wagner Grade IV ulcer with full-thickness skin loss and bone exposure
AA13	26	60	Male	Sole of the right foot	Wagner Grade IV ulcer with full-thickness skin loss and bone exposure
AA14	2	60	Female	Sole of the right foot	Wagner Grade IV ulcer with full-thickness skin involvement but without bone exposure
AA15	23	63	Male	Toes of the left foot	Wagner Grade IV ulcer with full-thickness skin involvement but without bone exposure
AA16	12	57	Male	Sole of the right foot	Wagner Grade IV ulcer with full-thickness skin involvement but without bone exposure
AA17	20	69	Male	Toes of the left foot	Wagner Grade IV ulcer with full-thickness skin involvement but without bone exposure

### 2.6 Cell proliferation assay

A cell proliferation assay was performed using the Cell Counting kit-8 (CCK-8; 40203 ES 76, Yeasen, Shanghai, China). HaCaT cells were seeded into 96-well plates at an appropriate density. Cells in the control and experimental groups were treated accordingly, while culture medium without cells served as a blank control. After incubation at 37 °C and 5% CO_2_ for 0, 24, 48, and 72 h, 10 μL of CCK-8 solution was added and incubated for another 3 h at 37 °C. Subsequently, the optical density (OD) of the cells was measured using a microplate reader (ELX800, BioTek, USA) at 450 nm. The formula for cell proliferation rate calculation is as follows: Proliferation rate (%) = [(experimental OD blank OD)/(control OD blank OD)] × 100%.

### 2.7 Flow cytometric analysis of cell apoptosis

To detect cell apoptosis, the Annexin V-fluor647/PI cell apoptosis detection kit (40304 ES 60, Yeasen, Shanghai, China) was used. HaCaT cells were seeded into the plates and treated as required. Treated and control cells were incubated with 5 μL of Annexin V-fluor647 for 15 min and 10 μL of propidium iodide (PI) reagent for 15 min in the dark. Prior to flow cytometry analysis, the cells were passed through a 40 μm cell strainer to remove aggregates. The level of apoptosis was then determined by flow cytometry (FACSCanto, BD, USA).

### 2.8 Wound-healing assay

The HaCaT cells were seeded into plates and cultured to 90% confluence. The cells were then treated without serum for 4 h. A uniform wound was created in the cell monolayer by scraping with a sterile 200 μL pipette tip. The cells were cultured in basal medium supplemented with 10% FBS (10091148, Gibco, USA), 100 μg/mL of streptomycin, and 100 U/mL of penicillin (SV30010, HyClone, USA) at 37 °C. Wounds were observed at 0 and 21 h within the scrape lines, and representative points were marked and photographed in three individual fields. Wound closure was detected using an inverted microscope (MF52-N, Mshot, China) at ×100 magnification. The areas of wound gaps were measured using the ImageJ software. The cell mobility rate was calculated using the following formula: Cell mobility rate (%) = [(0 h cell wound area − 21 h cell wound area)/0 h cell wound area] × 100%.

### 2.9 Library preparation and sequencing

RNA-seq assays were performed by Wuhan Ruixing Biotechnology Co., Ltd.^1^ Total RNA was extracted from HaCaT cells using TRIzol (Ambion) and purified by two phenol–chloroform extractions. Purified RNA was treated with RQ1 DNase (Promega, Madison, WI, USA) to remove DNA. The quality and quantity of further purified RNA were assessed by measuring the absorbance ratio at 260 nm/280 nm (A260/A280) using a SmartSpec Plus spectrophotometer (BioRad, USA). Agarose gel electrophoresis (1.5%) was performed to assess the integrity of the purified RNA. After mRNA was captured from total RNA using the VAHTS mRNA Capture Beads 2.0 kit (N402, Vazyme, China), the transcriptome library construction was performed using VAHTS Universal V10 RNA-seq Library Prep Kit (NR616, Vazyme, China). Briefly, mRNAs were captured with VAHTS mRNA Capture Beads 2.0, fragmented, and converted into double-stranded cDNA. Following end repair and A-tailing, the DNA fragments were ligated to adaptors. The ligation products were enriched by PCR, purified, quantified, and stored at −80 °C before sequencing. Using FASTX-Toolkit (version 0.0.13) to remove low-quality sequencing reads and adapter sequences from the raw sequencing data, the clean reads were then aligned to the reference genome using HISAT2 software. Uniquely mapped reads were used to calculate gene read counts and FPKMs. Differentially expressed genes (DEGs) were screened using DESeq2 (FC ≥ 3/2 or ≤ 2/3 and adjusted *p*-value <0.05). Pathway analysis was performed using KOBAS (version 2.0) (17).

### 2.10 iRIP-seq analysis

The reference genome for IP is human, with version GRCh38.p14. The IP-RNA sequencing libraries were constructed by Wuhan Ruixing Biotechnology Co., Ltd.^2^ Briefly, 100 ng RNA was fragmented and converted into double-stranded cDNA. Following end repair and A-tailing, the DNA fragments were ligated to VAHTS RNA Multiplex Oligos Set 1 for Illumina (N323, Vazyme, China). The ligated products were amplified, purified, quantified, and stored at −80 °C prior to sequencing. Strand specificity was maintained by incorporating dUTP during the second-strand cDNA synthesis, thereby preventing its amplification.

### 2.11 RIP-qPCR

Improved RNA immunoprecipitation assays were performed according to the previously described method. RIP-qPCR was performed on the ABI QuantStudio 5 (Thermo, USA), followed by denaturation at 95 °C for 10 min, 40 cycles of denaturation at 95 °C for 15 s, and annealing/extension at 60 °C for 1 min. Each sample had three technical replicates. Using the 2^−ΔΔCT^ method to analyze the mRNA level. Statistical comparisons were performed using the two-way ANOVA or the paired Student’s *t*-test using GraphPad Prism software (Version 8.0, San Diego, CA, USA).

### 2.12 Statistical analysis

An unpaired two-tailed *t*-test (between two groups) or an analysis of variance (ANOVA) (multiple groups) was performed on cell biology and qPCR data. Statistical significance was defined as a *p*-value of less than 0.05. Data are presented as the mean ± standard error of mean (SEM). Each experiment was conducted in at least three biological replicates.

## 3 Results

### 3.1 Abnormal expression of YWHAZ in DFU

**Figure 1:**
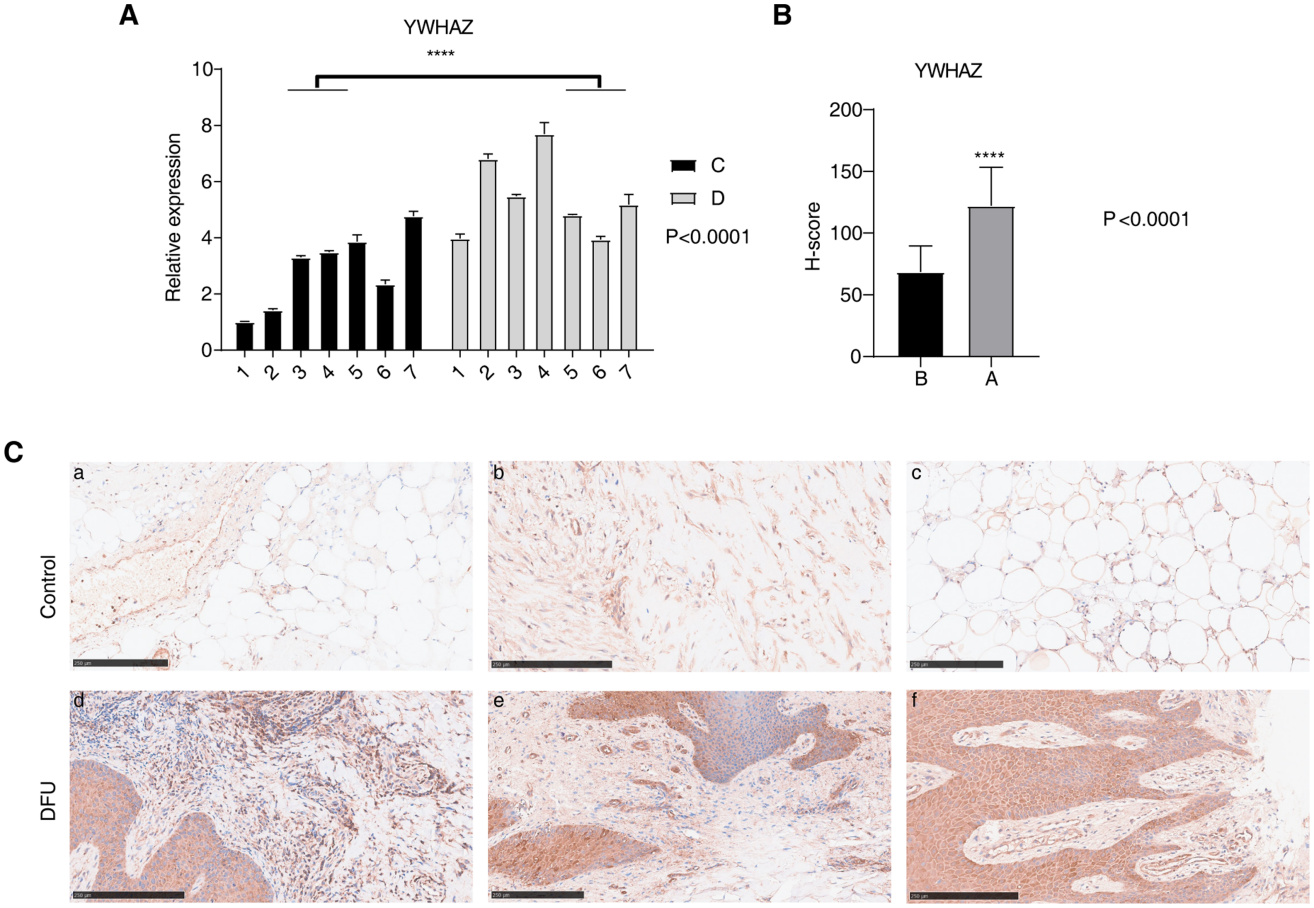
Detection of YWHAZ expression level. **(A)** Bar plot shows the expression level of YWHAZ mRNA, C: control skin tissue of a normal person, and D: ulcerated skin tissue of DFU patients. **(B)** Histochemistry score for YWHAZ in DFU and normal tissues, B: control skin tissue of a normal person, and A: ulcerated skin tissue of DFU patients. **(C)** Immunohistochemical staining for YWHAZ in DFU and normal tissues. (a–c) The control skin tissue of a normal person, and (d–f) ulcerated skin tissue of DFU patients. ****p-value <0.0001.

To identify DEGs in DFU tissues compared to NC tissues, this study analyzed published RNA-seq data (GSE134431) from ulcer skin tissues of six patients with non-healing diabetic foot ulcers (non-heal), and the normal skin tissues of eight patients with DFU as controls (Ctr), and performed DEG analysis. DEG analysis identified 1975 upregulated and 2,539 downregulated DEGs (Supplementary Figure S1A). Cluster analysis showed a clear separation among the three groups (Supplementary Figure S1B). GO functional enrichment analysis revealed that upregulated DEGs were associated with processes such as cornification, keratinocyte differentiation, establishment of skin barrier, membrane organization, and cell–cell adhesion, while downregulated DEGs were primarily linked to immune response (Supplementary Figure S1C). KEGG enrichment analysis demonstrated that the upregulated DEGs were mainly enriched in the GnRH signaling pathway, pathogenic *Escherichia coli* infection, endocytosis, and inflammatory mediator regulation of TRP channels, while the downregulated DEGs were mainly enriched in cell adhesion molecules (Supplementary Figure S1D). The above results suggest the dysregulation of the transcriptome profile with potential involvement in DFU. The identity and functions of abnormally expressed RBP genes in DFU remain unclear. In order to solve this problem, this study referenced the 2,141 reported RBPs (18) with the DEGs in RNA-seq, and 162 RBPs have been identified (Supplementary Figure S1E). YWHAZ emerged as the most significantly differentially expressed RBP in DFU-related datasets. Previous studies have shown that YWHAZ plays a crucial role in wound healing, inflammation regulation, and cell survival, suggesting its potential involvement in the pathological processes of DFU. Therefore, YWHAZ was selected for further study (Supplementary Figure S1F). The high expression of YWHAZ in DFU tissues was confirmed by qPCR (Figure 1A). Moreover, immunohistochemical staining showed strong YWHAZ expression in ulcerated skin tissue of DFU patients (Figures 1B,C).

### 3.2 YWHAZ knockdown regulates cell proliferation, migration, and apoptosis in HaCaT cells

**Figure 2:**
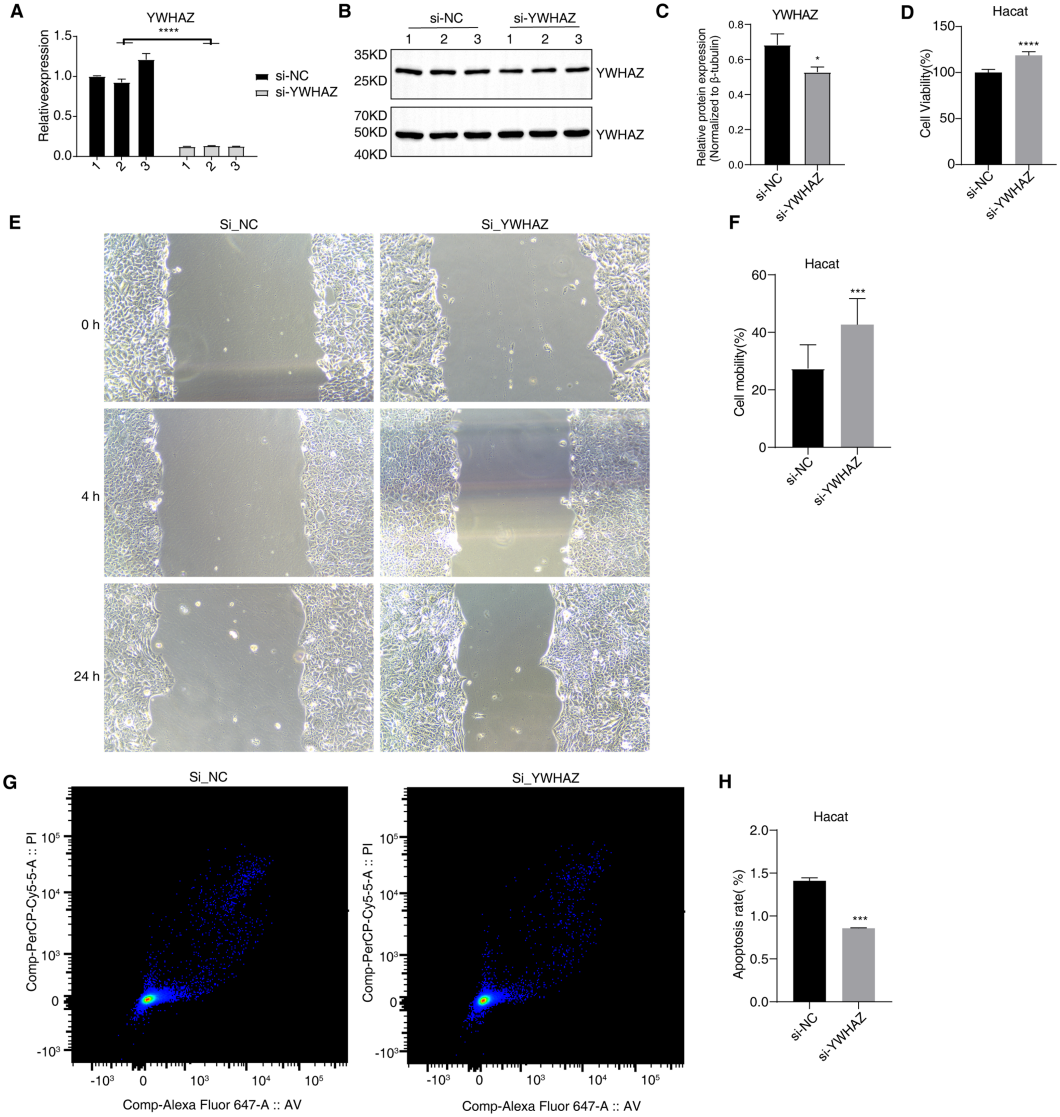
Effect of Si_YWHAZ_ on proliferation, migration, and apoptosis in HaCaT cells. **(A)** Bar plot shows the expression level of YWHAZ mRNA in Si_YWHAZ_ samples. **(B)** Western blot shows the expression level of YWHAZ protein in two groups. **(C)** Bar plot shows the quantitative result of western blot. **(D)** Bar graph displays the proliferation of HaCaT cells. **(E)** Wound-healing assay shows the migration of HaCaT cells. **(F)** Bar graph displays the migration of HaCaT cells. **(G)** Apoptosis rate in HaCaT cells detected by flow cytometry. **(H)** Bar graph displays the apoptosis of HaCaT cells. *p-value < 0.05, ***p-value <0.001, and ****p-value < 0.0001.

To explore the role of YWHAZ in DFU, YWHAZ was knocked down in HaCaT cells. HaCaT cells were transfected with a vector knocking down the YWHAZ gene or an empty vector. The knockdown efficiency was validated by a significant reduction in YWHAZ expression, as demonstrated by qPCR and Western blot analysis (Figures 2A–C). We found that YWHAZ knockdown significantly facilitated the proliferation and migration of HaCaT cells (Figures 2D–F). We used the Annexin V-fluor647/PI cell apoptosis detection kit to detect cell apoptosis. The results showed that the apoptosis level decreased after YWHAZ knockdown in HaCaT cells (Figures 2G,H). These findings collectively underscore YWHAZ’s role in regulating key cellular processes in keratinocytes.

### 3.3 YWHAZ knockdown changes the gene expression profiles of HaCaT cells

**Figure 3:**
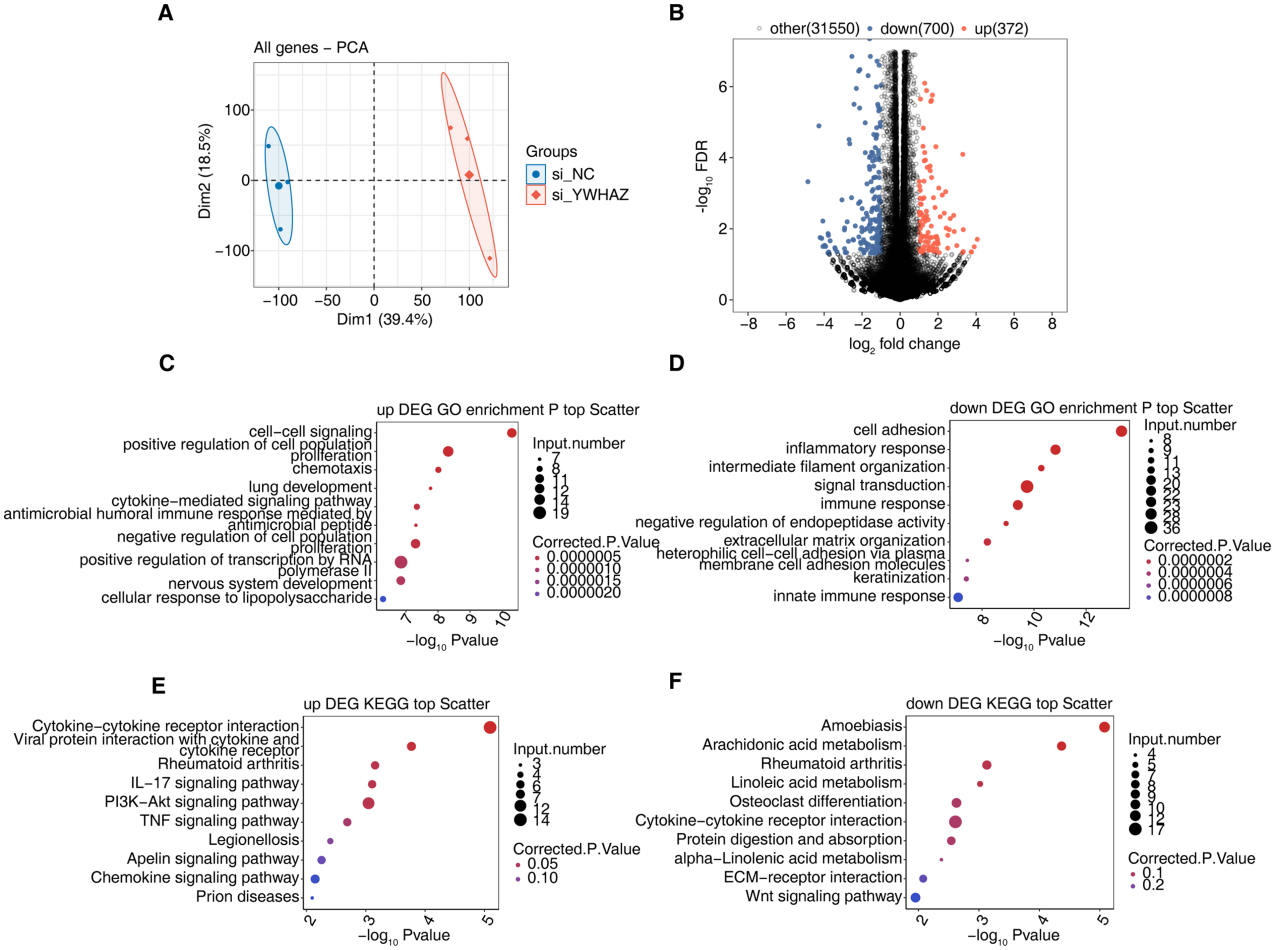
DEG screening and pathway analysis. **(A)** PCA based on the FPKM value of all detected genes. **(B)** Volcano plot shows the number of DEGs. **(C)** Bubble chart displays the most enriched GO biological processes for upregulated DEGs. **(D)** Bubble chart displays the most enriched GO biological processes for downregulated DEGs. **(E)** Bubble chart shows the most enriched KEGG pathways for upregulated DEGs. **(F)** Bubble chart shows the most enriched KEGG pathways for downregulated DEGs.

To explore the molecular mechanisms by which YWHAZ knockdown functions in HaCaT cells, the gene expression profiles of Si_YWHAZ cells and control cells were detected by RNA-seq. A total of six RNA-seq libraries were constructed and sequenced for Si_YWHAZ and control HaCaT cells, with three biological replicates for each group (Si_YWHAZ_1st, Si_YWHAZ_2nd, Si_YWHAZ_3rd, Si_NC_1st, Si_NC_2nd, and Si_NC_3rd). After removing low-quality reads, an average of 168.4 million clean reads per sample was obtained (Supplementary Table S1). Principal component analysis (PCA) based on all genes demonstrated a clear separation between Si_YWHAZ and NC samples (Figure 3A). A total of 1,072 DEGs were identified in Si_YWHAZ compared with NC tissues, with 372 upregulated and 700 downregulated DEGs (Figure 3B), indicating that YWHAZ knockdown extensively regulates gene expression in HaCaT cells. Detailed information for these DEGs, including fragments per kilobase of transcript per million mapped reads (FPKMs), fold changes, and description, is presented in Supplementary Table S2. These results indicated that YWHAZ knockdown significantly changed the transcript expression level of genes in HaCaT cells.

To elucidate the potential roles of DEGs, GO function analysis was performed to annotate all 1,072 DEGs. GO categories for biological processes showed that the upregulated DEGs were enriched for 48 GO terms, and the downregulated DEGs were enriched for 107 GO terms (Supplementary Tables S3, S4). It is notable that the upregulated genes in Si_YWHAZ cells were significantly enriched in the cell proliferation, such as *AREG*, *FOSL1*, *HAS2,* and *IL7R*, but the downregulated genes were significantly enriched in the cell adhesion, such as *LAMB3*, *SLAMF7*, *COL12A1,* and *ITGA5* (Supplementary Figure S2), which belong to the top 10 terms (Figures 3C,D). These results supported the YWHAZ knockdown affecting the proliferation of HaCaT cells. To further reveal the functional roles of these DEGs, KEGG pathway analysis was performed on 1,072 DEGs. KEGG pathway analysis revealed that the upregulated genes were enriched in pathways related to the cytokine–cytokine receptor interaction, viral protein–cytokine interaction, rheumatoid arthritis, IL-17 signaling, and TNF signaling(Figure 3E). Downregulated genes were enriched in rheumatoid arthritis and extracellular matrix (ECM)–receptor interaction (Figure 3F). These results implicate YWHAZ in the pathogenesis of diverse immune and inflammatory diseases through its regulation of inflammatory factor expression.

### 3.4 Identification of YWHAZ-interacting RNAs in DFU tissues

**Figure 4:**
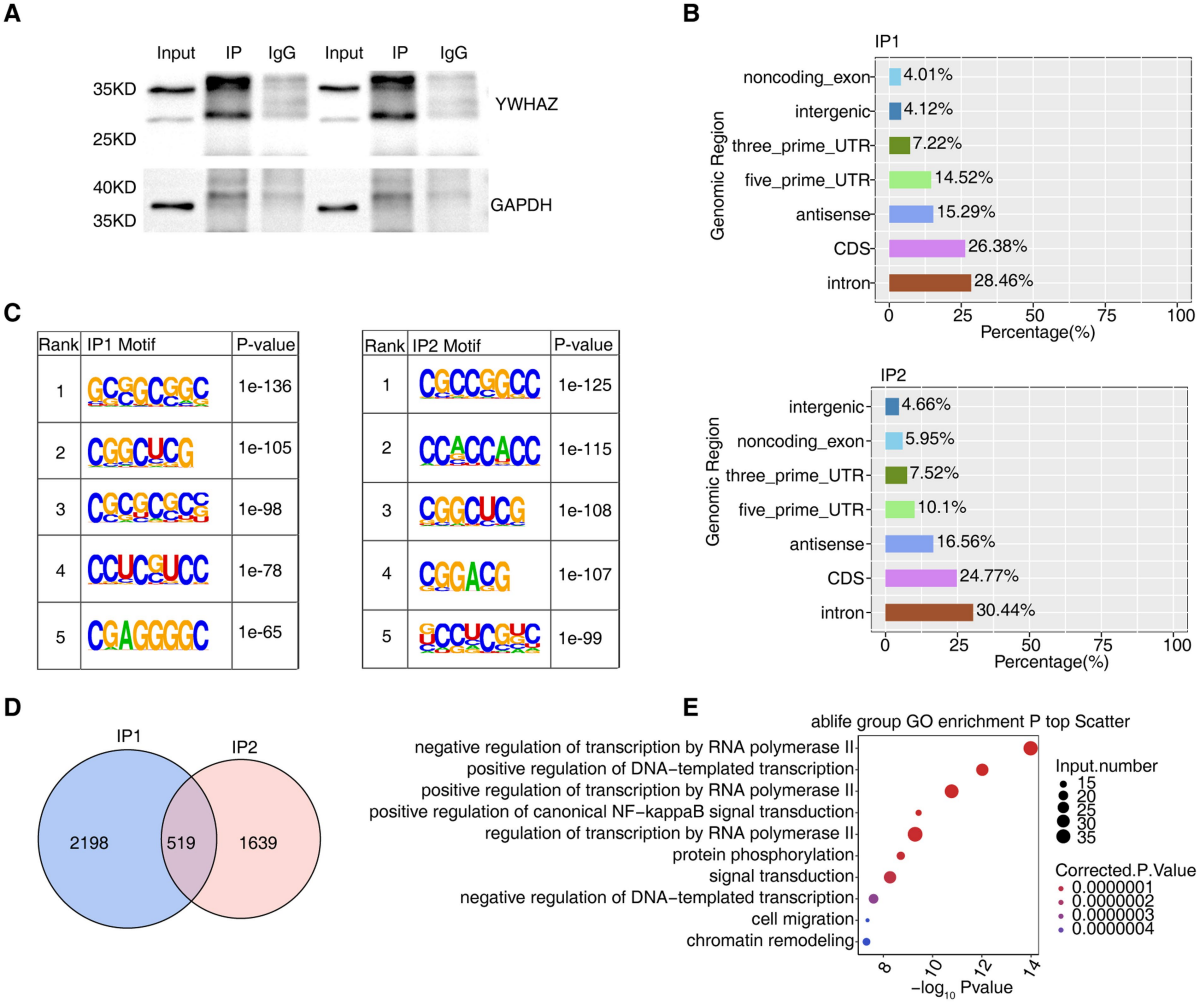
YWHAZ regulates mRNAs in DFU tissues. **(A)** Western blot displays the successful immunoprecipitation of YWHAZ in DFU tissues. **(B)** Bar graph shows the distribution of YWHAZ-bound peaks. **(C)** Motif analysis results show the enriched motifs from YWHAZ-bound peaks across the two biological replicates. **(D)** Venn diagram illustrates the overlap of YWHAZ-bound genes in IP1 and IP2. **(E)** Bubble chart displays the most enriched GO biological processes for YWHAZ-bound genes.

To delineate the interactions between YWHAZ and its RNA targets, this study used iRIP-seq coupled with comprehensive analyses to identify bound RNAs (Figure 4A). Four distinct cDNA libraries were generated, including two replicates for both YWHAZ_IP and Input samples. Moreover, the regions associated with YWHAZ-bound RNAs were analyzed and found that enrichment was mainly in the CD and intron region (Figure 4B). Enrichment in CDs indicates that the protein may play a central role in regulating the translation efficiency of mRNA (19). Furthermore, the enrichment in the intron region suggested that YWHAZ may bind to pre-mRNAs and affect RNA stability. Furthermore, the *de novo* motif analysis results (Figure 4C) showed that YWHAZ binds mainly to the GC-enriched region of RNA. The intersection of the two iRIP-seq datasets identified 519 genes associated with YWHAZ binding peaks (Figure 4D). These genes were observably involved in key pathways, such as negative regulation of transcription by RNA polymerase II, positive regulation of DNA-templated transcription, transcription by RNA polymerase II, canonical NF-kappaB signal transduction, and cell migration (Figure 4E). The results showed that binding of YWHAZ to *EPHA2* remains an important factor controlling cell migration. However, RIP-PCR results showed that *EPHA2* was not significantly enriched in the anti-YWHAZ samples. YWHAZ was bound to the exon regions of *HCFC1*, which is involved in the negative regulation of transcription by RNA polymerase II and positive regulation of DNA-templated transcription. This result was verified by RIP-qPCR (Supplementary Figure S3). Furthermore, these results indicate that YWHAZ-bound transcripts play important roles and may affect transcription in DFU tissues.

### 3.5 Identification of correlated genes with YWHAZ by RNA-seq and iRIP-Seq

**Figure 5:**
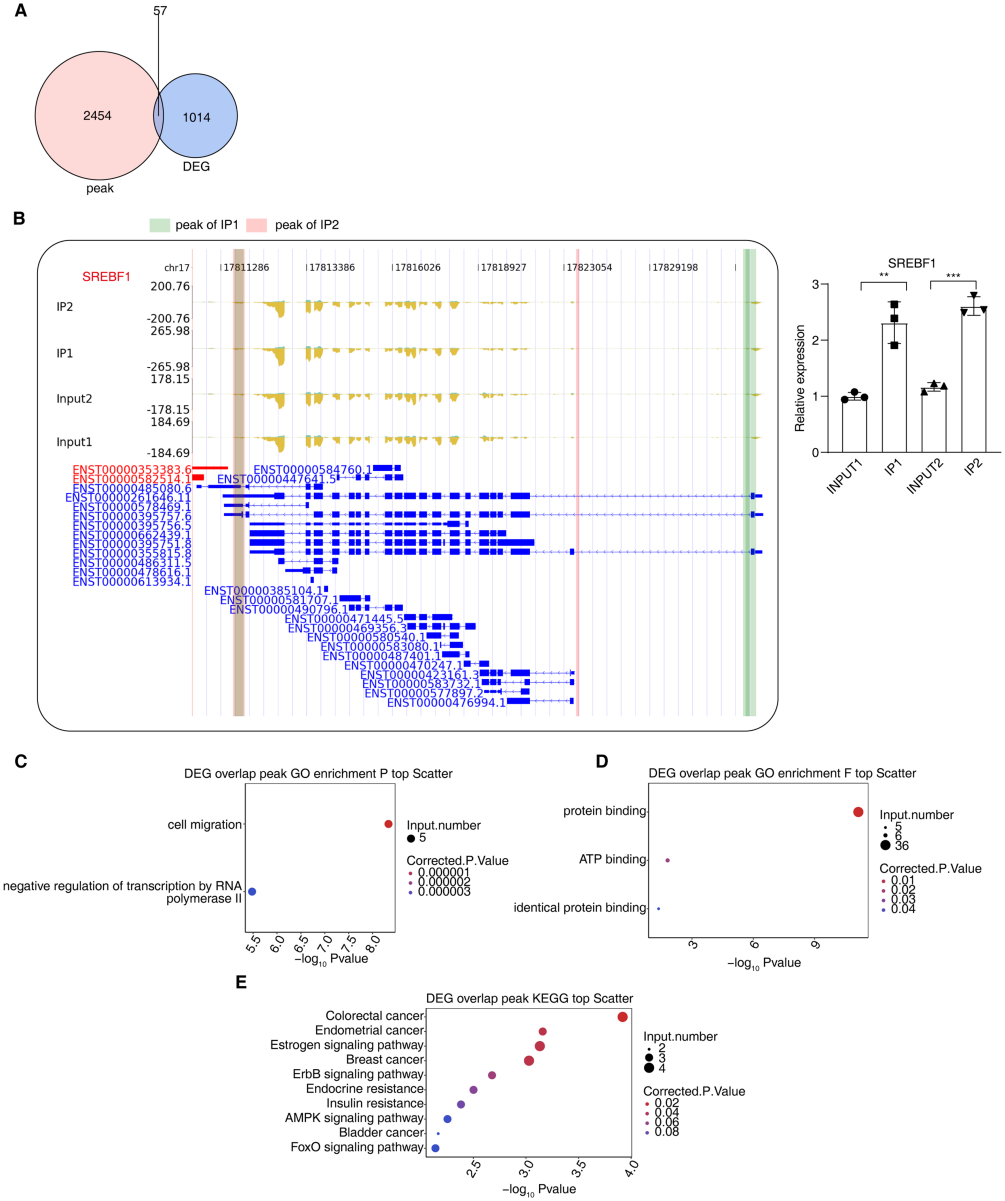
YWHAZ binds to target mRNAs in DFU tissues. **(A)** Venn diagram illustrates the overlap of genes between iRIP-seq and RNA-seq. **(B)** IGV-sashimi plot shows the distribution of *SREBF1* peak reads in iRIP-seq. **(C)** Bubble chart depicts the most enriched GO biological processes associated with the overlapping peak genes from iRIP-seq and RNA-seq. **(D)** Bubble chart depicts the most enriched GO molecular function associated with the overlapping peak genes from iRIP-seq and RNA-seq. **(E)** Bubble chart depicts the most enriched KEGG associated with the overlapping peak genes from iRIP-seq and RNA-seq.

An overlap analysis was performed between the DEGs identified by RNA-seq and the YWHAZ-bound genes to determine if they are distinctly correlated with YWHAZ. This study identified 57 DEGs that demonstrated this potential interaction (Figure 5A). Among them, 50 genes were downregulated, and 7 genes were upregulated. Notably, *SREBF1* was positively associated with T2D risk (20), showing binding signals with YWHAZ (Figure 5B). Furthermore, we confirmed binding between YWHAZ and *SREBF1* through RIP-PCR. Gene ontology (GO) analysis showed that *SREBF1* was significantly enriched in negative regulation of transcription by RNA polymerase II and protein binding (Figures 5C,D). Furthermore, the KEGG analysis revealed that *SREBF1* was enriched in insulin resistance, AMPK signaling, insulin signaling, and non-alcoholic fatty liver disease pathways (Figure 5E; Supplementary Table S5). These results further demonstrate that YWHAZ is implicated in DFU development. Specifically, the correlation observed between YWHAZ and SREBF1 suggests a potential regulatory relationship contributing to wound healing in DFU.

## 4 Discussion

RBPs are central mediators of post-transcriptional regulation and have been widely implicated in various pathophysiological processes (21). YWHAZ serves as a key regulatory hub that integrates multiple signaling pathways (22), and accumulating evidence has linked its elevated expression to several human diseases, including breast cancer, cardiofaciocutaneous syndrome, and colorectal cancer (23–25). However, the role of YWHAZ in DFU remains largely unexplored. In this study, a significant upregulation of YWHAZ in DFU tissues was initially identified through RNA-seq, which was further validated by RT-qPCR and immunohistochemistry. Functional experiments on HaCaT cells demonstrated that YWHAZ knockdown promoted cell proliferation and migration while suppressing apoptosis, supporting its regulatory role in DFU pathogenesis. To elucidate the underlying molecular mechanisms, iRIP-seq was performed, and the results were then integrated with RNA-seq data. This integrated approach revealed that YWHAZ selectively binds to 57 differentially expressed genes (DEGs), among which *SREBF1*—a gene linked to T2D risk—was particularly notable. These findings not only highlight the involvement of YWHAZ in DFU progression but also enhance the understanding of RBPs in chronic wound pathology, suggesting that YWHAZ is a potential molecular target for future therapeutic strategies.

During the proliferation phase, wound closure is primarily achieved through the activation of keratinocytes, which form a protective epithelial barrier (26). During the proliferation phase, pre-existing keratinocytes at the wound edge proliferate and migrate to cover the defect, while simultaneously rebuilding the basement membrane through protein secretion (27). Rapid proliferation and efficient migration of keratinocytes are critical steps in the wound healing process (28). The results observed that YWHAZ knockdown significantly enhanced the proliferation and migration of HaCaT cells. It is plausible that the high expression of YWHAZ serves as a “molecular brake” in DFU, thereby inhibiting keratinocyte-mediated repair. This finding provides a potential molecular explanation for the clinical observation of reduced epithelial cell migration at the wound edge and delayed healing in DFU.

Cell proliferation and adhesion are the key factors influencing DFU wound healing (29). DEGs identified following YWHAZ knockdown were significantly enriched in the cell proliferation and adhesion pathways. These results suggest that knocking down YWHAZ may affect DFU wound healing, potentially by regulating cell proliferation and adhesion. Following YWHAZ knockdown, the upregulation of *AREG*, *FOSL1*, *HAS2*, and *IL7R* was observed. Future studies should validate these DEGs by qPCR. Previous studies have shown that these genes can promote cell proliferation (30–33). The altered expression of *AREG*, *FOSL1*, *HAS2*, and *IL7R* in connection with YWHAZ implicates this gene set in the observed phenotype, suggesting a role for YWHAZ in regulating pathways relevant to wound healing and DFU development.

Although YWHAZ is known for its diverse molecular functions, its RNA-binding targets and characteristics have not been systematically investigated (34). This study reports the first comprehensive profile of YWHAZ-bound RNAs in HaCaT cells. iRIP-seq analysis revealed that YWHAZ interacts with a broad spectrum of mRNAs, with binding sites predominantly located in CD and intron regions. Furthermore, the ABLIFE algorithm showed that YWHAZ bound to GC-rich motifs, consistent with the binding characteristics of previously reported RBPs (35). Notably, among the YWHAZ-bound transcripts were mRNAs encoding regulators of transcription and cell migration, many of which have been linked to insulin receptor signaling pathways (36, 37). These results suggest the possibility that the previously uncharacterized RNA-binding activity of YWHAZ may play a crucial role in DFU progression.

The results of iRIP-seq showed that 57 DEGs were identified as binding partners for YWHAZ, while most of them were downregulated in DFU. The finding indicates that YWHAZ mainly functions as a post-transcriptional repressor in DFU. SREBF1 is a major insulin-induced transcription factor in the liver and visceral adipose tissue (VAT), regulating lipogenesis by binding to regulatory elements in genes, such as *LDLR*, thereby modulating cholesterol synthesis and uptake (38). Dong et al. (20) discovered that *SREBF1* was positively associated with type 2 diabetes (T2D) risk through genetic correlation analysis. Exogenous hydrogen sulfide (H_2_S) alleviates diabetic cardiomyopathy by promoting the ubiquitin-mediated degradation of *SREBP1* (39). Similarly, Nadiger et al. (40) reported that methylation of *SREBF1* in blood at baseline was associated with incident type 2 diabetes mellitus (T2DM)/hyperglycemia. YWHAZ may improve the expression of *SREBF1* by binding to its mRNA, thereby interfering with the lipid metabolism and the cellular energy supply required for DFU wound healing. The precise mechanisms underlying the interaction between YWHAZ and *SREBP1* warrant further investigation, potentially offering novel avenues for targeted treatment in DFU. We finally analyzed the functions of *SREBP1* and found that it was enriched in insulin resistance, the AMPK signaling pathway, the insulin signaling pathway, and non-alcoholic fatty liver disease. Thus, YWHAZ may contribute to DFU progression by modulating insulin levels. In summary, the regulatory role of YWHAZ in SREBF1 expression uncovered in this study not only provides a novel molecular perspective on keratinocyte dysfunction in DFU but also holds clear translational medical value. The YWHAZ–SREBF1 axis exhibits dual potential as both a predictive biomarker and a therapeutic target. Future research should validate this correlation in clinical samples and develop specific modulators, which may help in overcoming the current therapeutic bottlenecks in DFU.

Although this research provides new insights into the mechanism of YWHAZ in DFU, certain inherent limitations remain. While the siRNA-mediated knockdown approach used in this study suggests a potential role for YWHAZ in regulating HaCaT cell phenotypes, we acknowledge a technical limitation. The use of a single siRNA without complementary rescue experiments introduces the possibility of off-target effects. Nonetheless, the conclusions are primarily drawn from consistent, phenotype-specific changes observed following YWHAZ knockdown, which align with downstream molecular alterations. On the other hand, in immunohistochemistry experiments, ulcerated skin tissues from DFU patients were collected from the epidermis and upper dermis, whereas the control tissue was obtained from the dermis. Due to differences in cell populations between the epidermis and dermis, as well as increased adiposity in the dermal region, immunostaining patterns may vary. Thus, the study recognizes this as a limitation.

## 5 Conclusion

This study provides preliminary evidence that YWHAZ may be a dysregulated RBP in DFU. Initial experiments suggest that YWHAZ influences the phenotypes of HaCaT cells, which are associated with DFU development. We hypothesize that YWHAZ may enhance the expression of *SREBF1* by binding to it, thereby potentially perturbing lipid metabolism and the cellular energy supply crucial for wound healing in DFU. Overall, our findings indicate a potential foundational role for YWHAZ in DFU progression and offer early insights for future investigations into its regulatory mechanisms.

## Data Availability

All data generated or analysed during this study have been included in this published article and its supplementary information files. The datasets supporting the results of this article are available in the NCBI Gene Expression Omnibus and are accessible through GEO series accession numberGSE327462.

## Footnotes

1
http://www.rxbio.cc

2
http://www.rxbio.cc
