# Supplementary material for: RNA binding protein YWHAZ mediates specific mRNA translation and regulates cell proliferation and apoptosis in diabetic foot ulcer
Source: Front Med (Lausanne). 2026 May 7;13:1751279. doi: 10.3389/fmed.2026.1751279 (PMC13189950; doi:10.3389/fmed.2026.1751279)
Supplement: Supplementary file 7 [file Table_7.DOCX]

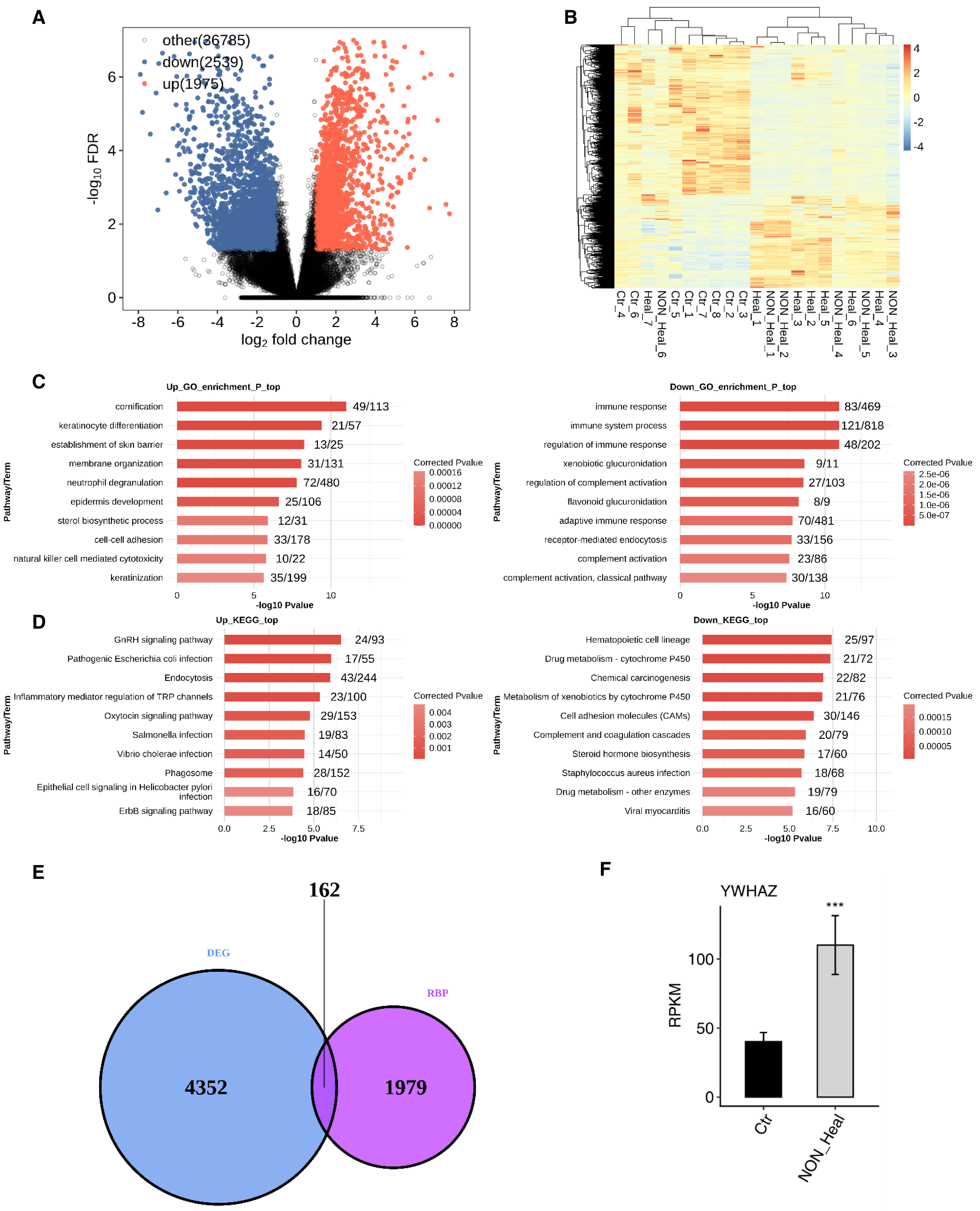


**Fig. S1** DEGs pathway analysis and RBP screening. **A** Volcano plot showing the number of DEGs. **B** clustering of DEGs. **C** Bar graph displaying the most enriched GO biological processes. **D** Bar graph displaying the most enriched KEGG. **E** Venn diagram illustrating the overlap of RBP genes from the reported 2141 RBP genes and the DEGs. **F** Bar graph displaying the expression patterns and statistical differences in RBP genes of DFU tissues and NC tissues in RNA-seq. The error bars indicate the mean±SEM. *** *p*-value<0.001.


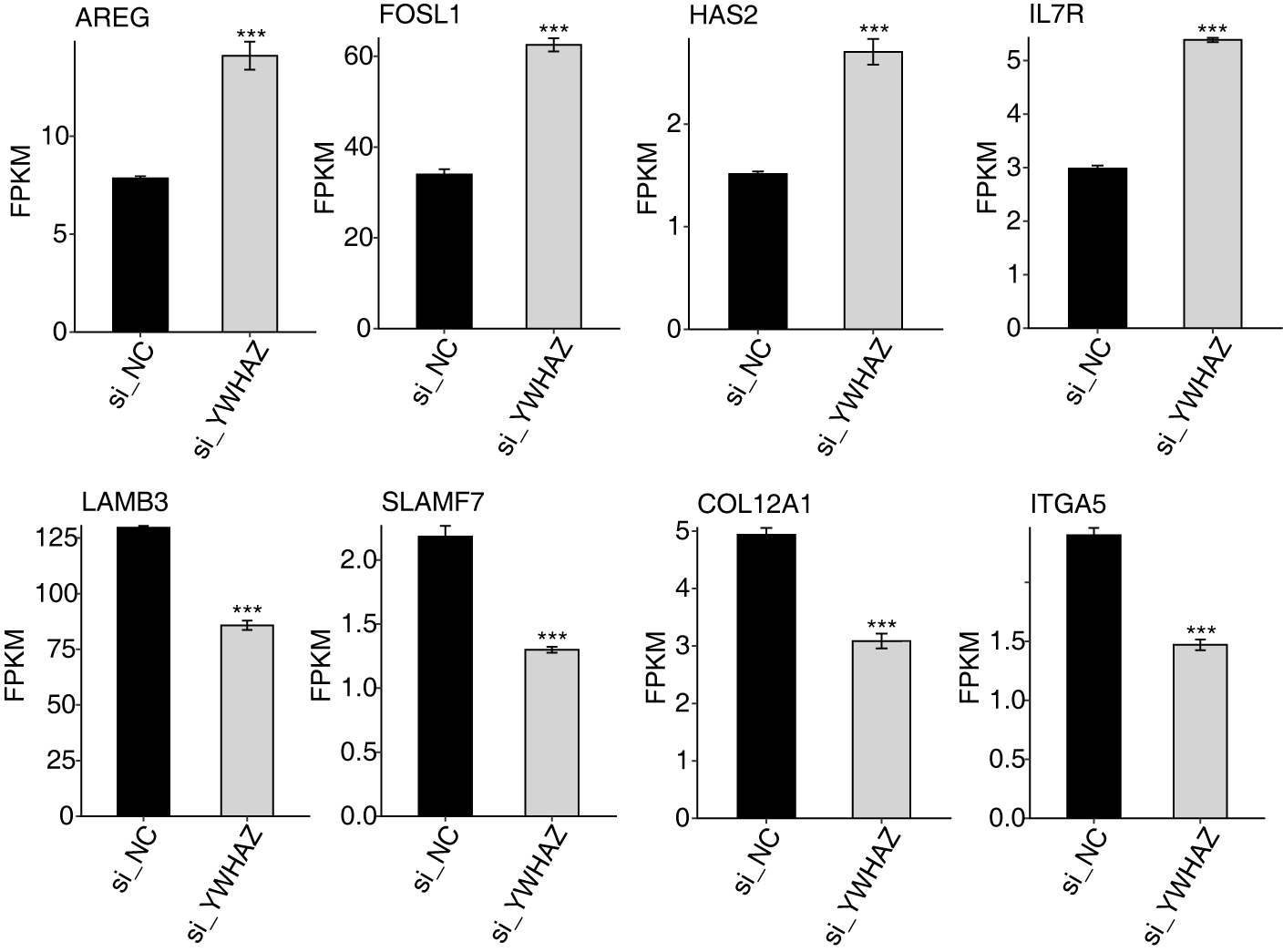


**Fig. S2** Bar graph displaying relative expression levels of selected DEGs measured by RNA-seq (FPKM).


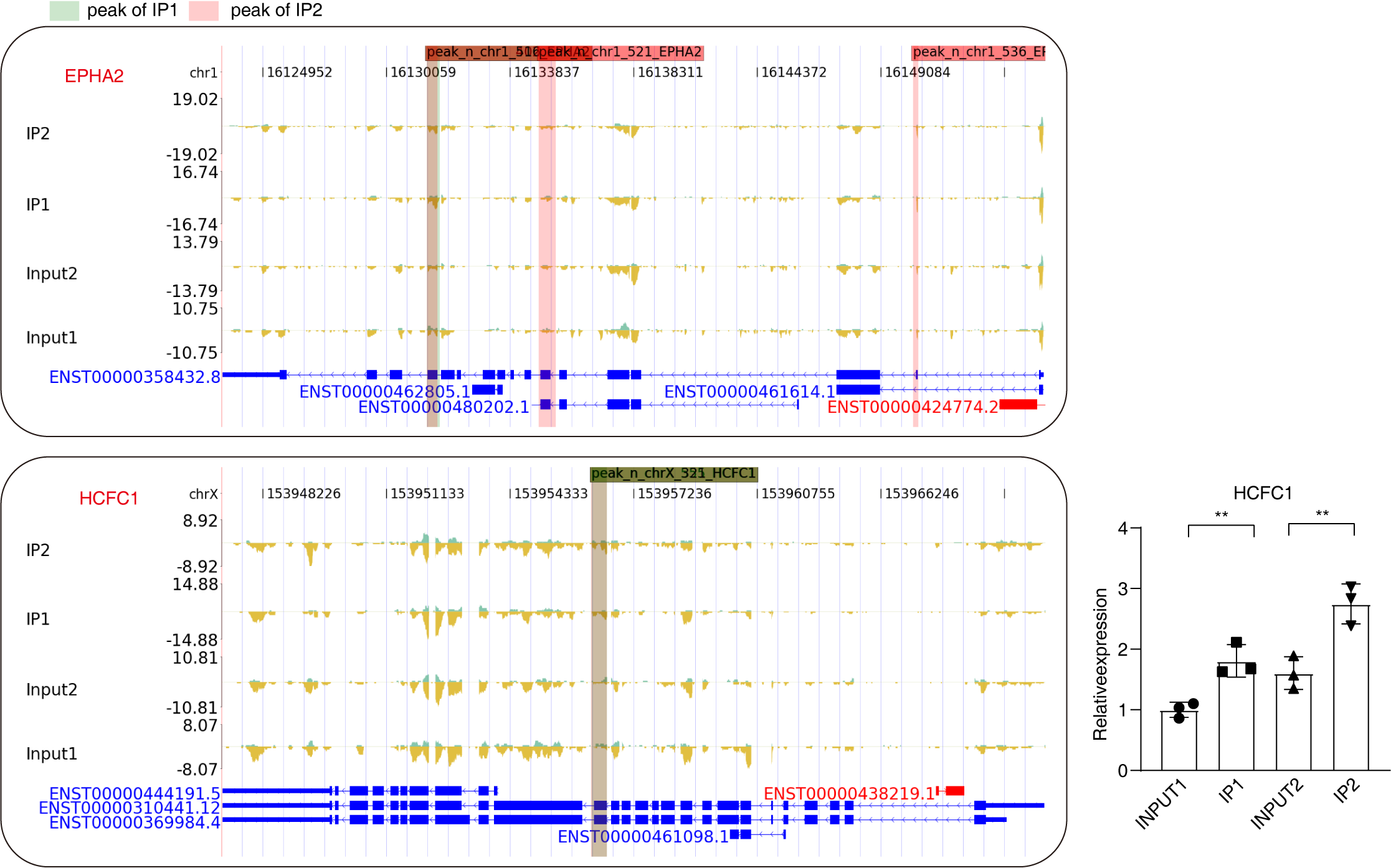


**Fig. S3** IGV-sashimi plot showing the distribution of YWHAZ-bound genes peak reads in iRIP-seq.
